# Dual Function pH Responsive Bispecific Antibodies for Tumor Targeting and Antigen Depletion in Plasma

**DOI:** 10.3389/fimmu.2019.01892

**Published:** 2019-08-09

**Authors:** Jan P. Bogen, Steffen C. Hinz, Julius Grzeschik, Aileen Ebenig, Simon Krah, Stefan Zielonka, Harald Kolmar

**Affiliations:** ^1^Department of Applied Biochemistry, Institute for Organic Chemistry and Biochemistry, Technische Universität Darmstadt, Darmstadt, Germany; ^2^Protein Engineering and Antibody Technologies, Merck KGaA, Darmstadt, Germany

**Keywords:** antibody discovery, bispecific antibodies, common light chain, recycling antibodies, yeast display, CEACAM5

## Abstract

Shedding of membrane-bound cell surface proteins, where the extracellular domain is released and found in the circulation is a common phenomenon. A prominent example is CEACAM5 (CEA, CD66e), where the shed domain plays a pivotal role in tumor progression and metastasis. For treatment of solid tumors, the presence of the tumor-specific antigen in the plasma can be problematic since tumor-specific antibodies might be intercepted by the soluble antigen before invading their desired tumor target area. To overcome this problem, we developed a generic procedure to generate bispecific antibodies, where one arm binds the antigen in a pH-dependent manner thereby enhancing antigen clearance upon endosomal uptake, while the other arm is able to target tumor cells pH-independently. This was achieved by incorporating pH-sensitive binding modalities in the common light chain IGKV3-15*01 of a CEACAM5 binding heavy chain only antibody. Screening of a histidine-doped light chain library using yeast surface display enabled the isolation of pH-dependent binders. When such a light chain was utilized as a common light chain in a bispecific antibody format, only the respective heavy/light chain combination, identified during selections, displayed pH-responsive binding. In addition, we found that the altered common light chain does not negatively impact the affinity of other heavy chain only binders toward their respective antigen. Our strategy may open new avenues for the generation of bispecifics, where one arm efficiently removes a shed antigen from the circulation while the other arm targets a tumor marker in a pH-independent manner.

## Introduction

Colorectal cancer (CRC) is the third most diagnosed cancer with approximately 10% of all diagnosed cancers. A specific and sensitive marker for colorectal and gastric carcinomas is the carcinoembryonic antigen-related cell adhesion molecule 5 (CEACAM5, CEA, CD66e) which is a membrane protein found on the surface of columnar epithelial and goblet cells of the colon (1). Detached tumor cells can survive the integrin-mediated anoikis by direct interaction of cell-bound CEACAM5 with Death Receptor 5, increasing the risk of metastatic development (2). During tumor progression, CEACAM5 is shed off the tumor cell surface and can be detected in the serum, where it is a clinically reliable marker for CRC diagnosis (3). Studies imply that the soluble CEACAM5 is a driving factor for metastatic development in the liver by stimulating kupffer cells to secret proinflammatory cytokines such as IL-10, IL-6, and TNF-α into the hepatic sinusoid (4–6). This in turn leads to the upregulation of cell adhesions proteins, which facilitate the arrest of circulating tumor cells. Liver metastasis is the main reason for CRC-related deaths (7).

Besides its pro-metastatic function, CEACAM5 in the blood stream can trap anti-CEACAM5 antibodies and thereby impede the direct targeting of the tumor (3). To circumvent that problem, recent developments aimed at generating CEACAM5 targeting antibodies that exclusively recognize CEACAM5 in its membrane-bound form but not the shed protein (8). Another member of the CEA family of highly related cell surface glycoproteins is CEACAM6, which is also a tumor target since aberrant expression leads to the development of human malignancies (9–11). A hallmark of some tumors is the simultaneous overexpression of both proteins (9, 10).

Even though monoclonal antibodies are a very important drug class, they can be limited in their efficiency and selectivity. As a consequence, numerous next generation antibody formats have been developed including those, where two different epitopes can be addressed by one single molecule. Bispecific antibodies (bsAbs) harbor two antigen binding sites and can therefore bind a single target biparatopically or different targets simultaneously aimed at mediating superior efficacy compared to the combination of two individual monospecific IgGs (12–14). In addition, bsAbs have been designed to bring different cell types in close proximity leading to the formation of immunological synapses, which is not possible with conventional IgGs (15).

A strategy, often pursued in recent decades to generate bispecific antibodies relies on protein engineering of the Fc part by incorporating an asymmetrical CH3:CH3 interface to force heterodimer formation. To this end, already in the 90ths of the last century the knob-into-holes technology was developed, where engineering of several residues within the CH3:CH3 interface forces heavy chain heterodimer formation thus allowing for the arrangement of two different antigen binding VH domains on a single antibody scaffold (16, 17). A plethora of alternative strategies for the generation of heavy chain heterodimers was developed over the years (18–20). These strategies successfully promoted pairing of cognate heavy chains but they do not address the light chain pairing problem. A straightforward solution of this problem is the use of common light chains, where the same light chain is used for pairing of the two different heavy chains (21). In this class of common light chain bispecifics, antigen binding is mainly or exclusively mediated by the heavy chain. Several strategies were described for the isolation of common light chain bsAbs that rely on synthetic human antibody libraries based on common light chains (22), immunization of transgenic rodents expressing a single common light chain (23) or the combination of a heavy chain repertoire with a predefined light chain followed by high throughput screening for binders (24).

**Figure 1:**
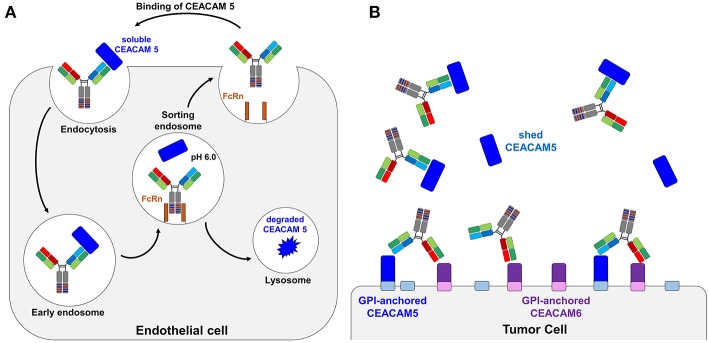
Schematic representation of a bispecific pH-responsive anti-CEACAM5 antibody that binds pH-independently CEACAM6. **(A)** Illustration of the bispecific SEEDbody as a recycling body in the periphery. The antibody together with its antigen is internalized by pinocytosis. In the acidic conditions of the endosome, the antibody releases CEACAM5 (blue) due to the pH-shift and gets recycled by the neonatal Fc receptor (orange). The antigen is degraded in the lysosome. **(B)** Depiction of the binding behavior of CEACAM5/CEACAM6 bispecific antibodies. The CEACAM5-specific heavy chain (CH1 and VH illustrated in blue) is binding to shed CEACAM5 (blue boxes). The CEACAM5-attached GPI anchor is depicted as a small box on the tumor cell surface in light blue. The CEACAM6-specific CH1-VH are depicted in red/orange and solely bind to the tumor surface attached CEACAM6 (depicted in violet, GPI-anchor in light violet).

Another next generation format is antibodies with pH-responsive binding modalities. For the efficient removal of target proteins from the blood stream, Igawa et al. proposed antibody engineering toward pH-dependent binding (25). Antibodies undergo a permanent recycling through endothelial cells via endocytosis and pinocytosis. Through binding to endosomal FcRn, the antibody is recycled and returned to the blood stream, even if the antigen is bound to the antibody. This process can prolong the half-life of the antigen *in vivo* and is known as antibody buffering (26). An elegant way to get rid of the antigen during this recycling process makes use of the fact that slightly acidic conditions prevail in the endosome. A pH-dependent antibody that has low affinity to its target antigen at acidic pH can release the bound antigen in the endosome which is eventually transported to the lysosome and degraded, while the antibody is recycled back to the circulation due to FcRn-binding (27) (Figure 1A). The cyclic process of antigen removal can be further enhanced by engineering the FcRn binding site of antibody Fc (28). To obtain pH-dependent binders, antibodies are generated that contain several histidine residues in their complementary determining regions (CDRs). Histidine is an uncharged amino acid at neutral pH but becomes positively charged at slightly acidic conditions. As a consequence, repulsive electrostatic forces may lower affinity to the target significantly (25, 29).

In this work, we describe a modular workflow with which we generated histidine-doped common light chain variants that are capable of pairing with CEACAM5-binding heavy chains and enable the pH-responsive binding to the antigen. To this end, we generated a histidine-doped light chain library paired it with the CEACAM5-binding heavy chain C5A (24) and enriched pH-responsive, CEACAM5-specific Fabs utilizing yeast surface display and fluorescence activated cell sorting (FACS). Exemplary proof of concept reformatting into an anti-CEACAM5/CEACAM6 bispecific format revealed an antibody, where one arm was binding its target in a pH-dependent manner, while the other remained pH-independent (Figure 1B).

## Materials and Methods

### Plasmids

The vectors used for yeast surface display of the heavy chain and the secretion of the common light chain were based on the pYD1 plasmid backbone (Yeast Display Vector Kit, version D, #V835-01, Thermo Fisher Scientific). The heavy chain encoding plasmid contained an ampicillin resistance marker, a tryptophan auxotrophic marker, the AGA2 signal peptide as well as the genes for the variable heavy chain domain (VH) and the constant CH1 domain of human IgG_1_. Besides a kanamycin resistance marker and a leucine auxotrophic marker, the light chain plasmid encoded the αMFpp8 signal sequence for soluble secretion of the VL and the CL domain (30). Gene expression of either plasmid was controlled via the galactose inducible promotor (*GAL1*). The His-doped light chain repertoire was integrated via homologous recombination into the light chain bearing plasmid after digestion with the restriction endonucleases *Pst*I-HF and *Sac*I-HF (NEB). For the expression of the Fab-constructs as one-armed SEEDbodies (oaSEEDbodies) or bispecific SEEDbodies, the genes for the light chain were subcloned into a pTT5 plasmid backbone (Expresso CMV based system, Lucigen) with ampicillin resistance and transfected into mammalian cells together with the respective heavy chain containing plasmids.

### Yeast Strains and Media

The *Saccharomyces cerevisiae* strain EBY100 [*MATa URA3-52 trp1 leu2*Δ*1 his3*Δ*200 pep4::HIS3 prb1*Δ*1.6R can1 GAL (pIU211:URA3*)] (Thermo Fisher Scientific) was transformed with the heavy chain containing plasmid. For the construction of the light chain library, the *S. cerevisiae* strain BJ5464 (*MAT*α *URA3-52 trp1 leu2*Δ*1his3*Δ*200 pep4::HIS3 prb1*Δ*1.6R can1 GAL*) (American Type Culture Collection) was utilized. Initially, strains were cultivated in YPD medium composed of 20 g/L peptone/casein, 20 g/L glucose and 10 g/L yeast extract supplemented with appropriate antibiotics (ampicillin 100 mg/L, kanamycin sulfate 75 mg/L or chloramphenicol 25 mg/L). The SD-CAA media for HC plasmid containing cells comprised 5.4 g/L Na_2_HPO_4_ and 8.6 g/L NaH_2_PO_4_ × H_2_O, 20 g/L glucose, 5 g/L ammonium sulfate, 1.7 g/L yeast nitrogen base (without amino acids), and 5 g/L bacto casamino acids. Since the heavy chain encoding plasmid contained a tryptophan auxotrophic marker whereas the light chain plasmid contained a leucine auxotrophic marker, the cultivation media for haploid or diploid cells was supplemented accordingly. Cultivating yeast cells on agar plates was performed in SD-CAA medium with the respective drop-out mix (Supplementary Table 1) supplemented with 7% agar-agar. For induction of Fab surface presentation, the diploidic yeast cells were cultivated in SG-CAA medium (composition same as for SD-CAA medium but instead of glucose galactose was used).

### Library Construction and Yeast Mating

The C5A and C6B heavy chain sequences as well as the IGKV3-15*01 common light chain coding sequence were described recently (24). Proceeding from the IGKV3-15*01 common light chain in the pYD plasmid, the CDR-L1 and CDR-L3 together with surrounding framework regions were PCR amplified utilizing primers encoding for two histidines in all possible CDR-L1 or CDR-L3 positions (Supplementary Tables 2, 3) in separate PCR reactions. In the subsequent SOE-PCR, these His-doped gene fragments were fused with wild type gene fragments, generating full-length light chain genes with incorporated histidines in the CDR-L1 or CDR-L3, respectively. In additional reactions, the CDR-L1 His-doped gene fragments were fused with CDR-L3 fragments carrying additional histidines utilizing SOE-PCR resulting in light chain genes encoding for two histidines in both CDRs (four histidines in total; Supplementary Figure 1). PCRs were performed utilizing Q5^®^ High-Fidelity polymerase (NEB): 98°C 30 s, 30 cycles of 98°C for 10 s, 68°C for 15 s and 72°C for 20 s, followed by a final elongation at 72°C for 20 s. The PCR products were cleaned up using the Promega Wizard® SV Gel and PCR Clean-Up System and subsequently pooled for library generation according to the protocol of Benatuil and coworkers (31). The pYD vector encoding the original IGKV3-15*01 light chain was digested utilizing the restriction enzymes *Pst*I-HF and *Sac*I-HF (NEB) and further purified form a 1% agarose gel utilizing Promega Wizard® SV Gel and PCR Clean-Up System kit. Overall 12 μg of DNA was utilized for electroporation and the resulting haploid yeast cell library was grown in SD-CAA-Leu medium.

In order to generate a diploid yeast library and to generate clones that are able to display a complete Fab molecule, EBY100 was transformed with the heavy chain vector encoding the C5A heavy chain and cultured in SD-CAA-Trp. 3 ×10^8^ cells of the light chain library and the equal number of heavy chain carrying yeast cells were mixed, centrifuged and resuspended in 150 μL YPD medium. Subsequently, 50 μL of the cell suspension was put on a pre-warmed YPD agar plate and incubated at 30°C over night. The next day, cells were washed of the agar plate and the resulting diploid library was grown in SD-CAA-Leu/-Trp medium.

### Library Sorting

For library sorting, the diploid yeast library was grown overnight in tryptophan and leucine deficient SD-CAA medium at 30°C and 180 rpm. Afterwards, cells were transferred to SG-CAA medium with a corresponding dropout mix at 10^7^ cells/ml followed by an incubation period of 1 day at 30°C. Cells were harvested by centrifugation and washed once in 1 mL PBS-B pH 7.4 [PBS + 0.1% (w/v) BSA]. The presentation of the Fab fragments on the yeast cell surface was verified utilizing the anti-human κ R-phycoerythrin (R-PE) goat F(ab')_2_ conjugate (Southern Biotech, diluted 1:75 in PBS-B pH 7.4). Cells were subsequently washed once with PBS-B at pH 7.4 and a second time either with PBS-B at pH 7.4 or pH 6.0, respectively. For target binding, yeast cells were incubated with recombinant human His-tagged CEACAM5 (R&D systems; 62.5 nM in PBS pH 7.4 or 6.0, respectively or phosphate-citrate buffer at pH 5.0). After a wash step with PBS-B at pH 7.4 or pH 6.0, cells were washed a second time at pH 7.4. All subsequent staining steps were performed at pH 7.4. For detection of target binding an anti-penta-His antibody (Qiagen, dilution 1:50) and an anti-mouse APC antibody (Invitrogen, diluted 1:50) were utilized (Supplementary Figure 2). All staining steps were performed on ice for 30 min (CEACAM5) or 15 min (labeling reagents), respectively, with a staining volume of 20 μL per 1 ×10^7^ yeast cells. After the final wash step in PBS-B, the cells were resuspended in PBS-B and analyzed in the BD Influx FACS Cell sorter (sortware 1.0.0.650). Yeast cells that were collected during the sorting process were transferred into SD-CAA medium and were incubated at 30°C and 180 rpm for 48 h before inoculating SG-CAA medium for subsequent sorting.

### Cloning, Expression, Purification of oaSEEDbodies and Bispecific SEEDbodies

After the identification of single clones displaying a pH-responsive Fab fragment, the plasmids were isolated utilizing the Zymoprep Yeast Plasmid Miniprep kit (Zymoresearch). The light chain genes were amplified using primers adding a 5' *EcoR*I site (*I CLC to pTT5, II CLC to pTT5*) and a 3' *BamH*I site (CLC to pTT5 rev) of the gene, as well as internal primers substituting an *EcoR*I site (*Fr3 EcoRI mut for/rev*) by a silent mutation (all oligonucleotides for cloning and reformatting are listed in Supplementary Table 3). The resulting PCR products were fused in a PCR resulting in a full-length light chain gene with overhangs for restriction. Amplifications were performed utilizing Phusion polymerase (NEB) at 98°C for 30 s, 30 cycles at 98°C for 10 s, 57°C for 15 s and 72°C for 45 s, followed by a final elongation at 72°C for 2 min. After subsequent restriction of the PCR products as well as the pTT5 destination vector with *EcoR*I-HF and *BamH*I-HF (NEB) inserts and the plasmid were ligated utilizing T4 DNA Ligase (NEB) over night at 16°C. The reaction mixture was directly transformed into *E. coli* DH5α and cells were subsequently plated on ampicillin containing DYT agar (16 g/L trypton/casein, 10 g/L yeast extract, 5 g/L NaCl, 7.5 g/L agar-agar). The plasmids from the resulting clones were sequenced at Microsynth Seqlab and positive single clones were picked and inoculated for plasmid preparation using Promega Midi Prep Kit (Promega).

For the determination of k_on_, k_off_, K_D_ values as well as the melting temperature, the constructs were expressed as one-armed SEEDbodies (oaSEEDbodies) as described recently (24). These antibodies consisted of three chains: a VH-CH1-CH2-SEED-AG chain, a CH2-SEED-GA chain and a light chain comprising VL and CL domains. IMAC purification was performed utilizing the C-terminal His6-tag of the AG-fragment to exclude GA homodimers (24).

For the soluble production of pH-responsive one-armed SEEDbodies, Expi293F cells were cultivated in 30 mL Expi293™ Expression Medium (ThermoFisher) and grown for 2 days at 37°C and 8.0% CO_2_ at 110 rpm. Transient transfections of Expi293F cells were performed at a cell density of approx. 2.7 ×10^6^ cells/mL, utilizing 15 μg of each plasmid with 120 μg polyethylenimine (PEI). To this end, plasmid and PEI were mixed and incubated in Expi293™ Expression Medium (Thermo Fisher) for 15 min before adding it dropwise to the cell suspension. After 24 h, the cell suspension was supplemented with 825 μL of aqueous 20% tryptone/casein solution. After 5 days of protein production, the cells were harvested at 3000 rpm for 3 min and the supernatant was filtrated with a 0.45 μm syringe filter and mixed 3:2 (v/v) with IMAC Binding Buffer (50 mM Tris/HCl pH 7.5). Protein purification was performed with an ÄKTA Start FPLC system in combination with a HisTrap HP column (GE Healthcare). After a 5 CV wash step with IMAC Binding Buffer, the POI was eluted with a linear gradient to 100% IMAC Elution Buffer (50 mM Tris/HCl pH 7.5, 500 mM imidazole). Fractions with the POI were pooled and used for subsequent purification via Protein A chromatography. To this end, 500 μL up to 5 ml of Protein A Binding Buffer (20 mM sodium phosphate, pH 7.0) were added and the sample was applied to an equilibrated Protein A HP column (GE Healthcare). Bound protein was eluted with 100% Protein A Elution Buffer (100 mM citrate, pH 3.0). 250 μL of Protein A Neutralization Buffer (1 M Tris/HCl, pH 9.0) was instantly added to 1 mL of Protein A eluate. The fractions were either dialyzed against PBS or buffer exchange was performed utilizing Amicon Ultra Centrifuge Filters (Amicon® Ultra-15 Centrifugal Filter Units, Merck Millipore, 3 kDa MWCO).

### Biolayer Interferometry

The binding kinetics were determined utilizing the Octet RED96 system (FortéBio, Pall Life Science). Measurements were performed at 30°C and 1000 rpm. All biosensors were incubated in PBS prior to utilization for at least 10 min. After determination of the baseline in PBS buffer for 10 s, the anti-human Fc biosensors or anti-human CH1 biosensors, respectively, were loaded with SEEDbodies at a concentration of 5 μg/ml in PBS for 120 s. Quenching of the biosensors was performed for 60 s in kinetic buffer (KB; PBS pH 7.4 or pH 6.0, respectively, or phosphate-citrate buffer pH5.0 + 0.1% Tween 20 + 1% BSA) prior to incubation of the sensors with 100 nM CEACAM5 or CEACAM6 in KB for 180 s. The dissociation of the antigen was measured for 300 s in KB at the respective pH. For each BLI experiment, a negative control was measured where the biosensor was incubated in KB instead of the respective antigen solution. This negative control was subtracted from all antigen containing samples. Data analysis was performed with FortéBio data analysis software 8.0 using a 1:1 binding model with Savitzky-Golay filtering. To ensure the stability of the pH-responsive light chains over multiple binding and release periods, anti-human CH1 biosensors were loaded with the C5A + CV2 oaSEEDbody and subsequently loaded with CEACAM5 at pH 7.4 in KB for 180 s. Afterwards, the dissociation at pH 5.0 was measured for 300 s. The association and dissociation steps were repeated six times.

### Thermal Stability Assay

The melting temperature of the purified protein was determined utilizing SyproOrange dye fluorescence. Therefore, the protein solution was diluted to 100 μg/ml with a 1:1,000 dilution of SyproOrange in a total volume of 40 μL and a temperature gradient was applied from 30 to 100°C with a gradient of 1°C/min. The measurement was performed with the BioRad 96CFX RT-PCR detection system. The calculation of the TM values was performed with the BioRad 96CTX RT-PCR software.

## Results

Davis and coworkers recently described a strategy for the generation of bispecific antibodies, where heavy chain heterodimerization is achieved by co-expression of Fc-engineered heavy chains that contain mutual exchanges of IgG/IgA segments (SEEDbody technology) (18, 32). These antibodies share a common light chain on both binding arms. One variant, C5A, was reported to bind CEACAM5 with subnanomolar affinity when paired with the germline-derived IGKV3-15*01 light chain (24).

To investigate, whether the common light chain that originally is not involved in target binding can be functionalized to mediate pH-dependent target binding, a subset of common light chain VL domains was generated, where CDR-L1 and CDR-L3 contain two histidines in all positional combinations. Since CDR-L2 is located further away from the heavy chain CDRs in comparison to CDR-L1 and CDR-L3, it was neglected for His-doping. CDR-L1 and CDR-L3 of the IGKV3-15*01 VL domain encoding sequence were His-doped separately by VL gene PCR amplification, in which 91 unique primers, each bearing two histidine codons were used (Supplementary Table 2). This resulted in 55 CDR-L3 and 36 CDR-L1 double His combinations. Finally, random combination of CDR-L1 and CDR-L3 variants resulted in 1980 light chain variants each of which containing two histidines at random positions of both CDR-L1 and CDR-L3. This led to a total of 2071 His-doped light chain variants. The His-doped gene fragments were inserted into a pYD1-derived yeast secretion vector via homologous recombination (strain BJ5464), resulting in a library of 7.5 ×10^5^ clones, indicating high coverage of all possible His combinations.

**Figure 2:**
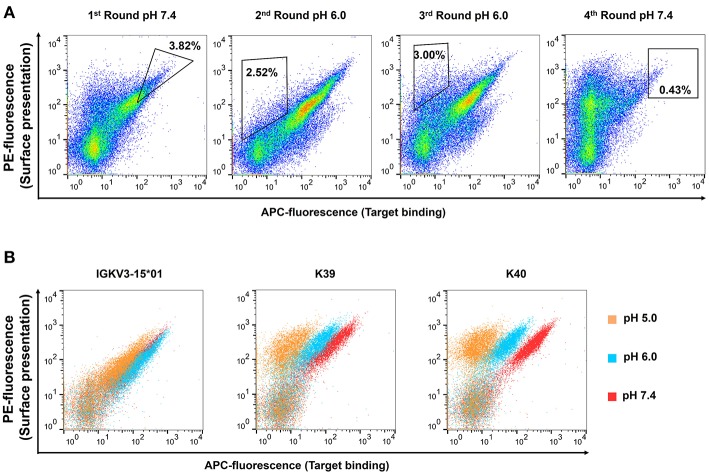
Consecutive FACS sortings for the enrichment of **(A)** pH-responsive CEACAM 5 binding Fab-fragments and **(B)** resulting single clones. Target binding is plotted on the x-axis (APC-fluorescence), surface presentation on the y-axis (PE-fluorescence). Each plot contains 50,000 events. Surface presentation was confirmed utilizing the anti-κ PE-antibody. Target staining was performed utilizing 62.5 nM CEACAM5, a anti-His mouse antibody and a anti-mouse APC-antibody. **(A)** In the first round, CEACAM5 binding Fab-fragments were enriched to deplete variants that were not able to bind CEACAM5 due to the histidine incorporation into the CDRs of the light chain. In the second and the third round, target staining was performed at pH 6.0 and yeast cells were sorted that displayed a Fab-fragment that does not bind to CEACAM5. For the fourth round, yeast cells were isolated that were able to display a CEACAM5 binding Fab at neutral conditions. Single clones were picked after this sorting round. **(B)** Overlay of plots from isolated pH-responsive single clones. The single clones were incubated with CEACAM5 at pH 5.0 (orange), 6.0 (blue), or 7.4 (red), respectively.

In the next step, 3 ×10^8^ cells of the light chain library were mated with 3 ×10^8^ EBY100 cells containing the CEACAM5 binding heavy chain variant C5A, resulting in diploid yeast cells capable of displaying the Fab fragment on the yeast cell surface via fusion to Aga2p (Supplementary Figure 2). With a mating efficiency of 17.2%, the final diploid library was subjected to FACS screening. To this end, CEACAM5 variants displaying yeast cells were incubated with His-tagged CEACAM5 and stained with an anti-penta-His antibody and an anti-mouse APC antibody. A control stain of the initial library in the absence of CEACAM5 showed no APC fluorescence, indicating that the anti-penta-His antibody does not recognize the His-doped CDR sequences (data not shown). To observe surface presentation of the full-length Fab construct, anti-Kappa PE F(ab′)_2_ was utilized. In the first sorting round, 3.82% of double positive yeast cells for display and CEACAM5 binding were enriched aimed at depleting light chain variants, where the His doping disrupted antigen binding (Figure 2A). In order to isolate pH responsive binders, the CEACAM5 stainings for the second and the third round were performed at pH 6.0. Gating for those rounds were applied to isolate yeast cells that showed surface presentation but lacked CEACAM5 binding. The final sorting round was performed at pH 7.4 to isolate pH-responsive single clones. All sorting rounds were performed with 62.5 nM CEACAM5.

**Figure 3:**
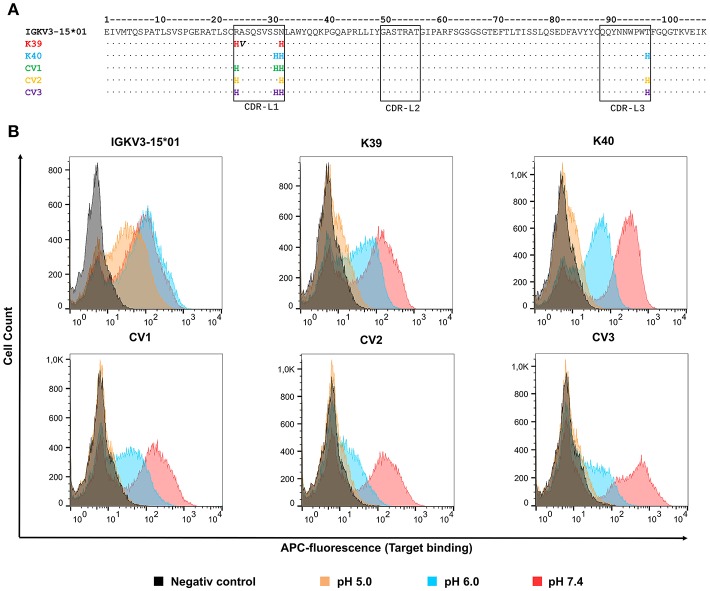
Sequences and FACS analysis of the pH-responsive, His-doped common light chains. **(A)** Sequence alignment of the parental IGKV3-15*01 common light chains with the isolated pH-responsive light chains K39, and K40 as well as the permutated light chain variants CV1, CV2, and CV3. Histidines in CDR3 and CDR1 are highlighted in the respective light chain color. Non-histidine mutations are highlighted in bold black. The numbering refers to the Chothia numbering scheme. **(B)** Histograms of yeast cell single clone carrying the respective light chain in combination with the C5A heavy chain on the yeast surface. All cells, except the negative control which was incubated in PBS, were incubated with 62.5 nM CEACAM5 at their respective pH-value, and subsequently all cells were washed with PBSB at pH 7.4, and further labeled with anti-His and anti-mouse-APC. For each experiment 50,000 yeast cells were recorded.

50 isolated yeast clones were analyzed for CEACAM5 binding at pH 6.0 compared to pH 7.4. While all clones showed strong binding to CEACAM5 at neutral conditions, 3 clones displayed reduced affinity at acidic pH (Figure 2B). Subsequent sequence analysis of the pH-dependent binders resulted in two unique pH-responsive variants, referred to as K39 and K40. Both variants showed significantly reduced binding signal at pH 6.0 and pH 5.0 (Figure 2B). K39 contained two histidines in the CDR-L1 and no His in CDR-L3, whereas K40 contained three histidines, two in CDR-L1 and unexpectedly only one in the CDR-L3 (Figure 3A). The absence of a second histidine within the CDR-L3 probably originates from homologous recombination events during library generation by gap repair. Both clones shared an N32H exchange within the CDR-L1 and displayed moderate pH-dependent binding (Figure 3B). To further improve the pH-dependence in antigen binding, the histidine patterns of both isolated single clones were combined, resulting in the combinatory variants (CV) CV1, CV2, and CV3 (Figure 3A).

FACS analysis confirmed the pH-responsive binding behavior of the permutation variants, since incubation at pH 5.0 completely abolished the binding signal (Figure 3B). Incubation of CEACAM5-bound cells at pH 6.0 showed similar results. As expected, the original IGKV3-15*01 common light chain domain that lacked any His residues in CDR-L1 and CDR-L3 showed no differences in CEACAM5 release at pH 6.0 in comparison with pH 7.4 and only a slightly decreased binding at pH 5.0.

All five variants together with wildtype VL sequence were reformatted as one-armed SEEDbodies (oaSEEDbodies) to determine the binding characteristics to CEACAM5 as a soluble protein. To this end, the respective light chain gene was cloned into the recipient pTT5-VL-CL plasmid. For expression, 8.1 ×10^7^ Expi293F cells were transfected with 15 μg of pTT5-VL-CL, pTT5-VH-CH1-CH2-CH3(SEED-AG) and pTT5-CH2-CH3(SEED-GA), respectively. The purification was performed in a two-step workflow. First, the supernatant was purified after 5 days of protein production via IMAC utilizing the C-terminal His6-tag on the SEED-AG chain to ensure the purification of heterodimeric SEEDbodies. After dialysis, the SEED-containing solution was subjected to Protein A chromatography.

**Figure 4:**
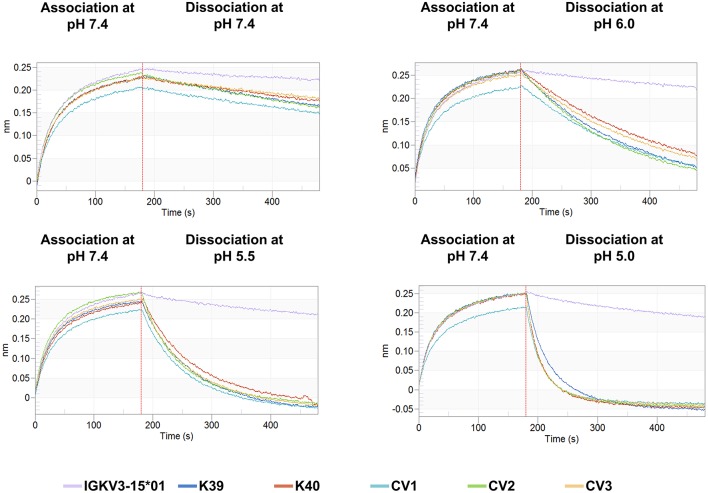
BLI sensorgrams of CEACAM5 binding to the pH-responsive clones K39, K40, CV1, CV2, and CV3 as well as for the parental non-pH-responsive IGKV3-15*01 immobilized on AHC tips. The association was performed with 100 nM CEACAM5 at pH 7.4 in KB. The dissociation was performed in KB at the respective pH value. The depicted lines are colored according to the respective light chain data.

The binding kinetics of the different CEACAM5 binding variants were determined using the FortéBio Octet96red BLI system. To this end, the oaSEEDbodies were loaded onto anti-human-Fc biosensors (AHC tips), quenched in kinetics buffer and the association was measured in 100 nM CEACAM5 solution at pH 7.4, as it resembles the pH-value of serum. The dissociation was performed at pH 7.4, pH 6.0, pH 5.5 and pH 5.0 in KB buffer, respectively to simulate the pH-shift of the antibody-antigen complex within the sorting endosome (Figure 4). Similar approaches were performed by Schröter et al. (29) and Igawa et al. (27).

The association rates of the engineered variants were nearly identical with the parental antibody, demonstrating that the histidines in the CDRs do not interfere with target binding at neutral conditions (Table 1). Nevertheless, the His-doped variants exhibited a lower K_D_ value at physiological conditions due the slightly accelerated dissociation. This phenomenon was also observed by Schröter and coworkers, where pH-responsive Adalimumab variants showed a 10 to 24-fold reduced K_D_ value compared to the parental antibody (29). The pH-responsive light chain variants from this work displayed a reduced the K_D_ in a range between 2.8- and 4.6-fold at pH 7.4 compared to the IGKV3-15*01 light chain.

**Table 1:** Binding kinetic parameters and T_M_ of IGKV3-15*01 and all pH-responsive light chain variants binding to CEACAM5.

**Antibody**	**pH 7.4**			**pH 6.0**	**pH 5.5**	**pH 5.0**	**K_**dis**_ ratio** **(pH 6.0) vs.** **IGKV3-15**	**K_**dis**_ ratio** **(pH 5.5) vs.** **IGKV3-15**	**K_**dis**_ ratio** **(pH 5.0) vs.** **IGKV3-15**	**Tm (**°**C)** **pH 7.4**	**Tm (**°**C)** **pH 6.0**
	**K_**D**_ nM**	**K_**on**_** **(×10^**−6**^)** **M^**−1**^ s^**−1**^**	**K_**dis**_** **(×10^**−4**^)** **s^**−1**^**	**K_**dis**_** **(×10^**−4**^)** **s^**−1**^**	**K_**dis**_** **(×10^**−4**^)** **s^**−1**^**	**K_**dis**_** **(×10^**−4**^)** **s^**−1**^**					
IGKV3-15*01	0.90	0.264	2.38	3.68	6.63	8.71	1	1	1	66.5 ± 0.7	65.0 ± 0.5
K39	4.16	0.238	9.89	50.7	156	353	14	24	41	66.7 ± 0.3	65.3 ± 0.3
K40	3.12	0.245	7.65	37.6	121	437	10	18	50	66.5 ± 0.0	65.3 ± 0.8
CV1	4.11	0.235	9.68	45.4	176	416	12	27	48	66.5 ± 0.5	65.0 ± 0.5
CV2	4.14	0.279	11.5	55.5	167	450	15	25	52	66.5 ± 0.5	64.8 ± 0.4
CV3	2.55	0.249	6.36	40.4	152	447	11	23	51	66.8 ± 0.3	65.3 ± 0.3

*The binding affinity is listed for association and dissociation at pH 7.4 with both k_on_ and k_off_. The k_off_ values for the different dissociation pH-values are also listed. The ratios of the dissociation constant at pH values <7.4 in comparison to k_dis_ at pH 7.4 are shown on the right together with the dissociation ratios under the same conditions*.

At pH 6.0 all engineered variants showed a 10–15-times elevated dissociation rate compared to the IGKV3-15*01 wild type light chain. When the dissociation was performed at pH 5.5, the dissociation was accelerated by the factor 18–27 and at pH 5.0 by the factor 41–52, clearly demonstrating the pH-responsive binding modalities of all variants that were in the range of previously published pH-responsive antibodies (27). In contrast, the parental antibody only exhibited a 4-times faster dissociation at pH 5.0 compared to neutral conditions (Figure 4, Table 1).

To exclude the possibility that the dissociation of CEACAM5 is due to pH-induced denaturation of the Fab-fragment, thermostability of all constructs was measured at neutral and acidic conditions. The resulting T_M_ values showed no significant difference between neutral and acidic conditions, indicating that the stabilities of the antibodies are pH-independent (Table 1).

**Figure 5:**
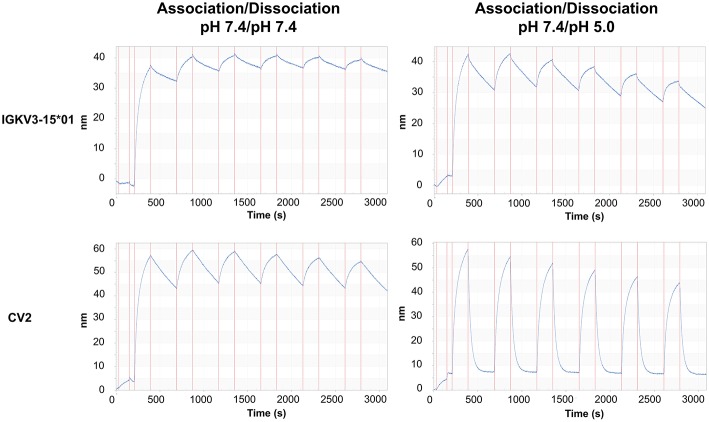
Sensorgrams of the periodic association and dissociation of CEACAM5 to immobilized oaSEEDbody comprising C5A and either IGKV3-15 or CV2. On the left panel, association and dissociation of CEACAM5 to the immobilized oaSEEDbodies was performed at pH 7.4. On the right, the dissociation was performed at pH 5.0. Each association step is immediately followed by a dissociation step. All experiments were performed with 100 nM CEACAM5.

Since CV2 exhibited the strongest change in the dissociation rates compared to the parental antibody, it was chosen for further experiments. To effectively reduce the concentration of an antigen in serum, a recycling antibody must be able to bind and release its antigen multiple times throughout its lifetime. To investigate whether the variant CV2 is suitable for the usage as a recycling antibody, its mode of action was simulated by performing consecutive association and dissociation cycles. Therefore, the antibody was loaded onto anti-Fab biosensor tips. Association of CEACAM5 was again performed at pH 7.4, which would correspond to serum pH. The dissociation was either performed at pH 5.0 to simulate the cellular uptake and undergoing the transportation to the sorting endosome, or at pH 7.4 (Figure 5). As a control, the parental antibody was treated identically. The IGKV3-15*01 comprising antibody bound tightly to the antigen and only a minor dissociation was observed at pH 7.4 within the measurement time frame of 5 min, which was only slightly increased at pH 5.0. In contrast, the CV2 displayed strong binding to CEACAM5 at pH 7.4 slow dissociation at pH 7.4 but fast dissociation at pH 5.0 over six repeated cycles of association and dissociation, although the binding capacity decreased over time, likely due to dissociation of the CV2 antibody from the anti-Fab biosensor (Figure 5). Nevertheless, these experiments showed that the modular approach of His-doping the common light chain that is not directly involved in the antigen binding can lead to highly pH-responsive variants, suitable for the usage as a recycling antibody.

To verify that the one arm CV2 SEEDbody retains its ability for tumor cell binding, we analyzed binding on COLO 205 cells by FACS (24, Supplementary Figure 3). High affinity binding was observed at pH 7.4 and reduced binding at pH 5.0. Unexpectedly, tumor cell binding was retained at pH 6.0 despite the fact that CV2 releases the soluble shed target already at this pH (Figure 4) which indicates that the microenvironment on the cell surface influences pH-dependent SEEDbody binding.

**Figure 6:**
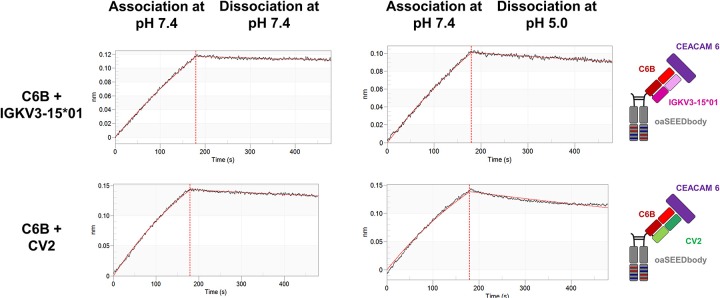
Biolayer interferometric measurements of the CEACAM6-binding C6B VH paired with CV2. C6B-derived oaSEEDbodies were loaded onto anti-Fab tips. The association of CEACAM6 was performed at pH 7.4 with a CEACAM6 concentration of 100 nM, the dissociation at pH 7.4 or pH 5.0, respectively, (black line). The fit (1:1 binding model with Savitzky-Golay filtering) for the K_D_ determination is depicted in red.

For use as bispecifics, it would be required that the His-doped light chain variant retains its capability to act as a common light chain when paired with other heavy-chain only binders. To investigate this aspect, the CEACAM6 binding C6B heavy chain was further investigated for CV2 pairing (24). BLI measurements of a C6B/CV2 oaSEEDbody showed antigen binding with a negligible difference in K_D_ (8.8 vs. 7.7 nM) compared to C6B/IGKV3-15*01 (Figure 6, Table 2).

**Table 2:** Binding kinetic parameters of the C6B heavy chain in combination with IGKV3-15*01 and CV2.

**Antibody**		**pH 7.4**			**pH 6.0**	**pH 5.0**	**K_**dis**_ ratio** **pH 6.0/7.4**	**K_**dis**_ ratio** **pH 5.0/7.4**	**K_**dis**_ ratio** **(pH 5.0) vs.** IGKV3-15*01
		**K_**D**_ nM**	**K_**on**_ (×10^**−4**^) s^**−1**^**	**K_**dis**_ (×10^**−4**^) s^**−1**^**	**K_**dis**_ (×10^**−4**^) s^**−1**^**	**K_**dis**_ (×10^**−4**^) s^**−1**^**			
C6B	IGKV3-15*01	8.77	0.02	1.58	1.90	3.61	1.2	2.3	1.0
	CV2	7.73	0.03	2.62	1.42	7.68	0.5	2.9	2.1

*The binding affinity K_D_ is shown for association and dissociation at pH 7.4, the k_dis_ is also listed for the dissociation at pH 6.0 and pH 5.0. The ratios of the dissociation constant at pH values <7.4 in comparison to k_dis_ at pH 7.4 are shown on the right together with the dissociation rates under the same conditions*.

**Figure 7:**
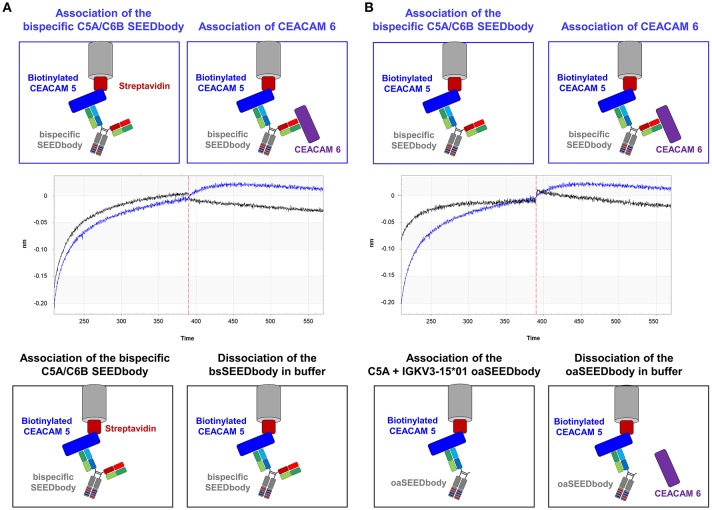
Validation of simultaneous binding of CEACAM5 and CEACAM6 by the bispecific C5A/C6B SEEDbody comprising the CV2 light chain. **(A)** Immobilization of biotinylated CEACAM5 on streptavidin tips and subsequent association of the bispecific C5A/C6B SEEDbody. In an additional step, CEACAM6 is associated (blue sensorgram), or the tip is incubated in PBS buffer (black sensorgram). **(B)** After the immobilization of biotinylated CEACAM5 on streptavidin tips, the bispecific C5A/C6B SEEDbody (blue sensorgram) or the C5A oaSEEDbody (black sensorgram) was added. Both tips were transferred to a CEACAM6 containing solution in an additional association step. Association steps are separated via a vertical red dashed line in the sensorgrams.

Finally, a bispecific SEEDbody was generated containing the C5A heavy chain in one arm and the C6B heavy chain in the other, both paired with the CV2 light chain. BLI measurements were performed to investigate the ability of the bispecific molecule to bind both antigens. After the loading step with biotinylated CEACAM 5 onto streptavidin tips, the SEEDbody was loaded and subsequently, CEACAM6 was added in the last association step. The bispecific antibody was able to bind both antigens specifically. As control, a C5A based oaSEEDbodies was utilized in order to exclude nonspecific interactions with CEACAM6 (Figure 7).

## Discussion

Most membrane-bound proteins shed their ectodomains to some degree (33). Wang and coworkers divided the therapeutic monoclonal antibodies (mAbs) into three categories based on the characteristics of their targets: (I) membrane-bound cell surface target protein with minimal shedding; (II) membrane-bound cell surface target protein that sheds its extracellular domain, and (III) circulating soluble targets, as e.g., growth factors and cytokines (34). Shedding of a pharmacological target from the surface of tumor cells, thus resulting in a soluble target that can also bind a therapeutic antibody, is a common phenomenon that may complicate cancer therapy (35). Whether target shedding positively or negatively affects the potency of a targeting molecule within a solid tumor remains a matter of debate (35, 36).

For shed targets, antibody-mediated antigen accumulation was observed (28) due to the fact that clearance of tight-binding antibody-antigen complexes is much slower than that of the free antigen. This problem has recently been overcome by recycling and sweeping antibodies, which can actively eliminate soluble antigens from circulation via pH-responsive endosomal target release followed by FcRn mediated recycling (27, 28). No efforts were made yet to use pH-responsive recycling antibodies for therapy of solid tumors, since it might be expected that due to extracellular acidification of the tumor tissue with pH values of 6.5 and lower antibody affinity is drastically reduced in the tumor microenvironment (37–39). Conceptually, this problem can be overcome using bispecific antibodies, where one arm targets tumor cells pH-independently, while the other arm mediates binding and removal of shed antigen via FcRn recycling in combination with receptor-mediated endocytosis (Figure 1B). To the best of our knowledge, no bispecific recycling antibodies were described, where one arm is engineered to enable the antibody to bind to an antigen in plasma and dissociate from the antigen in the endosome (after which the antigen undergoes lysosomal degradation), and the other mediates pH-independent target binding.

In recent years, a plethora of bispecific antibodies has been developed for various disease indications. Prominent applications are the recruitment of specific effectors of the immune system to target tumor cells or adjustment of binding specificity due to interaction with two different surface antigens (40, 41). To overcome the platform-associated issue of light chain pairing, the concept of common light chain bispecifics was developed thus facilitating antibody generation and purification (22, 23).

To obtain pH-responsive common light chain antibodies, we started from C5A, a human heavy chain that is able to bind CEACAM5 when paired with the IGKV3-15*01 light chain (24). We generated a light chain sublibrary that contained two histidines in either CDR-L1, CDR-L3, or in both. After four FACS-assisted sorting rounds, it was possible to identify clones K39 and K40 showing a pH-responsive binding behavior. Interestingly, K39 showed an additional A25V mutation besides the two histidines in CDR-L1 which was likely a result of PCR mutagenesis. The sequence of K40 also differed from the designed library composition by comprising only one histidine in CDR-L3 instead of two or none. These findings were not considered crucial for compromising the initial library quality since sequence analysis after library construction revealed the desired mutation pattern (data not shown).

In comparison with the IGKV3-15*01 light chain, where only a minor decrease of binding was detectable at pH 5.0, K39 and K40 showed significant reduced binding to CEACAM5 at pH 5.0 and also pH 6.0. Based on K39 and K40, permutated variants CV1, CV2 and CV3 were generated with different histidine combinations in order to further enhance the pH-dependence of CEACAM5 binding. Indeed, CV2 and CV3 showed further reduced antigen binding at acidic pH when displayed on the cell surface of yeast. To further elucidate their binding behavior, one-armed SEEDbodies were generated from these five variants. Biolayer interferometry clearly demonstrated that the association at neutral conditions is not negatively influenced by the histidine substitutions in the light chain CDRs. This is most probably due to the fact, that the IGKV3-15*01 light chain is not involved in the antigen binding (24).

In line with FACS analysis, K39 showed the least binding difference at different pH values whilst also comprising the least number of histidines (2 histidines in CDR-L1). The variants CV2 (3 histidines in total) and CV3 (4 histidines in total) showed the strongest pH-responses. These observations corroborate the finding of Murtaught and coworkers who reasoned, that the pH-dependency is elevated with increasing the number of ionizable groups (42). Interestingly, the CV2 variant showed a slightly higher k_dis_-value compared to the CV3 variant at pH 6.0 and pH 5.0, where the only difference is a single histidine at position 31. This indicates that the “dual histidine” motif might not be as favorable as supposed earlier (42). The variant CV3 showed higher k_dis_-values compared to CV1, which differs in the presence of His97 in CDR-L3. These findings hint to the importance of His97 for the pH-responsive binding modalities.

Since the CV2 variant showed the strongest pH-dependence in antigen binding, multiple bind and release cycles were performed using this CLC to investigate its suitability for the usage as a recycling antibody. The dissociation was performed at pH 5.0 to simulate the conditions in the sorting endosome over a course of 5 min. *In vivo* the sorting endosome takes about 8–15 min to recycle the cargo back into the plasma, underlining the suitability of CV2 as a recycling antibody (43).

As a proof of concept, it was verified that a His-doped common light chain can be utilized for the generation of bispecific antibodies able to bind one antigen pH-independently, while the second target is bound in a pH-responsive manner. In a first step, it was shown that the CV2 light chain can pair with the C6B heavy chain and bind pH-independently to CEACAM6. Following the generation of a C5A/C6B bispecific SEEDbody comprising the CV2 light chain, we demonstrated that this bsAb is able to bind both targets simultaneously. This further underlines the suitability of the concept of His-doped common light chains as a platform to engineer pH-responsive antibodies for mono- and bispecific antibody formats.

## Concluding Remarks

Several bispecific formats for CEACAM5 targeting were reported for cancer immunotherapy over the years such as an anti CEACAM5/CD3 BITE (MEDI-565/AMG 211) (44) or the head-to-tail 2:1 T cell bispecific antibody for treatment of CEA-positive solid tumors (45, 46) and others (47–49). pH-responsive binding has not been implemented yet in CEACAM5 targeting antibodies. Moreover, this type of bispecifics may be particularly useful for efficient removal of soluble targets that act as growth factors or suppress the induction of antitumor immunity such as TGF-β or IL10 (50) in combination with tumor cell binding or checkpoint inhibition. In conclusion, bispecific sweeping antibodies open new avenues for cancer treatment but will require sophisticated animal models to investigate their advantage over conventional formats. With the simple strategy described here, technical hurdles have been overcome on the road to novel sweeping antibody formats, but there is a long way to go for an in-depth examination of their potency in animals and eventually in humans.

## Data Availability

The raw data supporting the conclusions of this manuscript will be made available by the authors, without undue reservation, to any qualified researcher.

## Author Contributions

JB, SH, and HK conceived and designed the experiments. JB, SH, and SK performed the experiments. JB, SH, JG, SK, and HK analyzed the data. JG, AE, and SZ gave scientific advice. JB, SH, SK, and HK wrote the paper.

### Conflict of Interest Statement

SK and SZ were employed by Merck KGaA. The remaining authors declare that the research was conducted in the absence of any commercial or financial relationships that could be construed as a potential conflict of interest.
