# Supplementary material for: Dual Function pH Responsive Bispecific Antibodies for Tumor Targeting and Antigen Depletion in Plasma
Source: Front Immunol. 2019 Aug 9;10:1892. doi: 10.3389/fimmu.2019.01892 (PMC6697062; doi:10.3389/fimmu.2019.01892)
Supplement: Supplementary file 1 [file Data_Sheet_1.docx]

Supplementary Material

## 1 Supplementary Figures





**SUPPLEMENTARY FIGURE S1** | **Schematic illustration of the cloning strategy to generate a common light chain library in yeast with an elevated content of histidines**. The backbone of the pYD-VL-CL plasmid is shown in light gray, the sequences of the framework regions in dark gray, the CDR L1 in red, the CDR L2 in blue and the CDR L3 in green. Primers and their respective orientation are illustrated as arrows. Histidine substitutions in the CDR-L1 are shown as blue asterisks, in the CDR-L3 as red asterisks. PCR reactions are numbered. The amplified full-length genes (illustrated in blue frames) carry purple overhangs at the 5’ end and dark green overhangs at the 3’ overhang for subsequent gap-repair. Overhangs for SOE-PCRs are illustrated as dotted lines. All sequences are orientated 5’🡪3’.


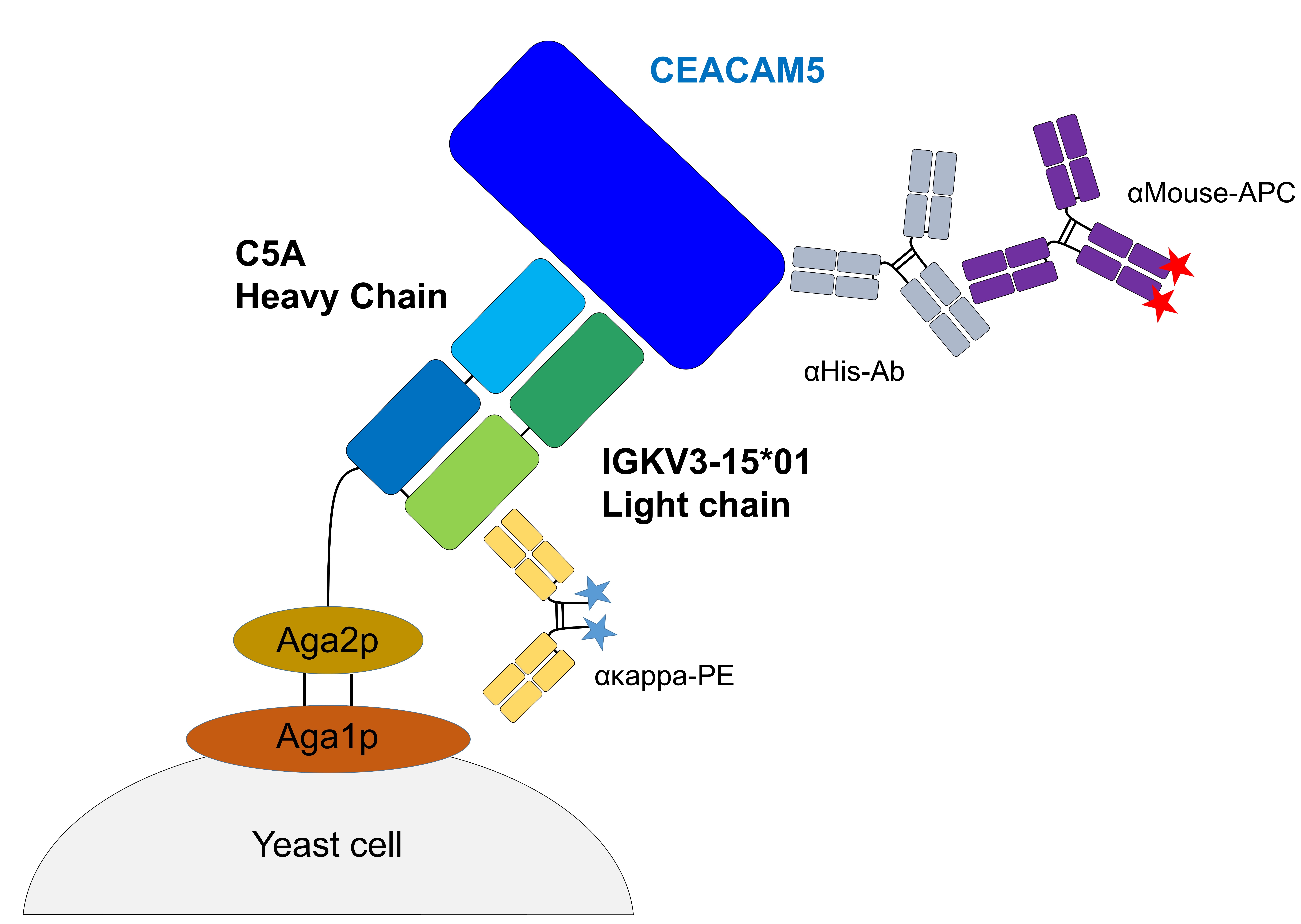


**SUPPLEMENTARY FIGURE S2** | **Schematic depiction of yeast surface display for the isolation of pH-responsive, CEACAM5 specific Fab variants**. The VH-CH1 domains (bright or dark blue, respectively) were N‑terminally fused to the Aga2p protein (brown) via a (GGGS)_4_ linker, while the Aga2p protein was connected to the Aga1p membrane protein (orange) via two disulfide bridges. The corresponding light chain (bright or dark green, respectively) was covalently bound to the heavy chain via a cystine. The surface presentation of the Fab was verified by staining with a ακappe-PE F(ab’)_2_ antibody (yellow). The binding to CEACAM5 (royal blue) was detected via an αHis antibody (grey) and a secondary αMouse-APC antibody (violet).

**
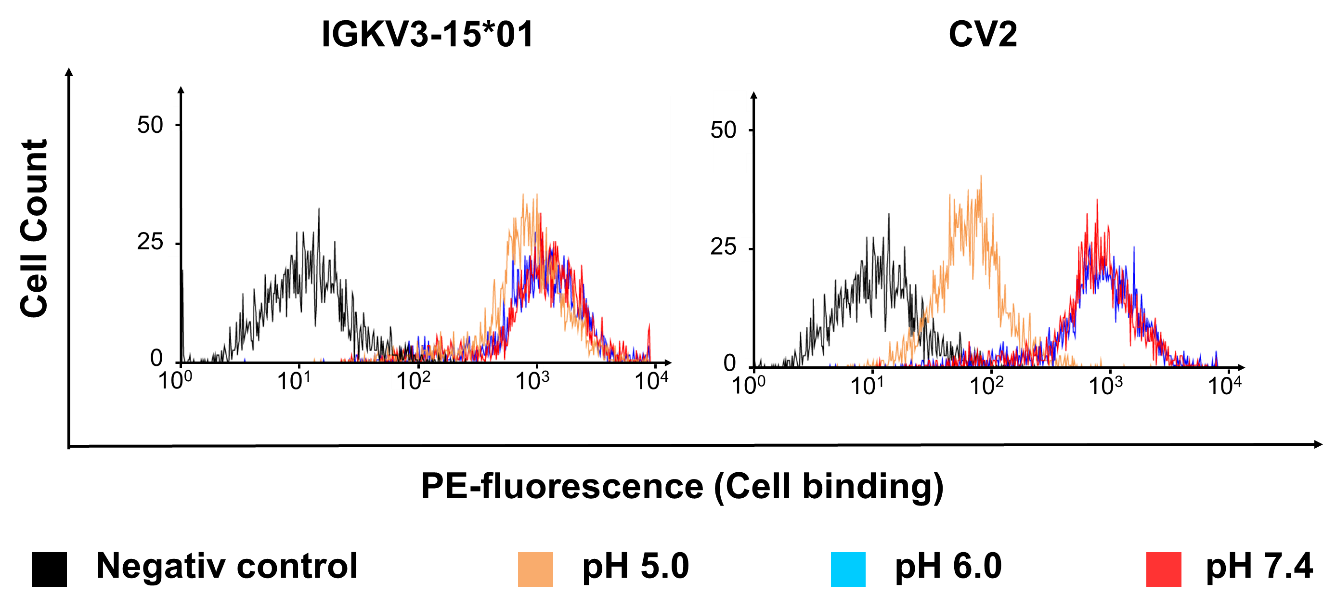
**

**SUPPLEMENTARY FIGURE S3** | **Cell binding of oaSEEDbodies comprising C5A and IGKV3‑15*01 or CV2 at different pH-values.** Around 4 × 10^5^ COLO 205 cells in 20 μl were stained with 3 µg of C5A-based oaSEEDbodies at different pH-values. For pH 7.4 and pH 6.0 PBS was used, while phosphate-citrate buffer was utilized at pH 5.0. Cell staining was performed as previously described by Krah and coworkers (24). Binding of the oaSEEDbodies was verified utilizing an anti-human Fc PE-antibody. Target binding is plotted on the x-axis (PE-fluorescence), cell count on the y-axis.

# Supplementary Tables

**SUPPLEMENTARY TABLE S1** | Amino acid composition of drop-out media.

| **Substance** | **Concentration in media (mg/mL)** |
| --- | --- |
| L-Adenine hemisulfate | 40 |
| L-Arginine | 20 |
| L-Aspartic acid | 100 |
| L-Glutamic acid | 100 |
| L-Histidine | 20 |
| L-Lysine | 30 |
| L-Methionine | 20 |
| L-Phenylalanine | 50 |
| L-Serine | 375 |
| L-Threonine | 200 |
| L-Tryptophan | 40 |
| L-Tyrosine | 30 |
| L-Valine | 150 |
| Uracil | 20 |

**SUPPLEMENTARY TABLE S2** | Oligonucleotides for the incorporation of histidines into the IGKV3-15*01 light chain. Mutations were incorporated either in the CDR-L1 or CDR-L3, respectively. The numbering refers to the Chothia numbering scheme. Mutagenesis sites are highlighted.

| Name | | | | Nucleotide sequence (5' --> 3') |
| --- | --- | --- | --- | --- |
| Position of histidine incorporation | **Orientation** | **CDR** | **Template** |  |
| 24/25 | rev | CDR1 | pYD-VL-CL | GTTTTTGTTGATACCAAGCCAAGTTAGAAGAAACGGATTGAGA**ATGATG**ACAAGACAATGTAGC |
| 24/26 | rev | CDR1 | pYD-VL-CL | GTTTTTGTTGATACCAAGCCAAGTTAGAAGAAACGGATTG**ATG**AGC**ATG**ACAAGACAATGTAGC |
| 24/27 | rev | CDR1 | pYD-VL-CL | GTTTTTGTTGATACCAAGCCAAGTTAGAAGAAACGGA**ATG**AGAAGC**ATG**ACAAGACAATGTAGC |
| 24/28 | rev | CDR1 | pYD-VL-CL | GTTTTTGTTGATACCAAGCCAAGTTAGAAGAAAC**ATG**TTGAGAAGC  **ATG**ACAAGACAATGTAGC |
| 24/29 | rev | CDR1 | pYD-VL-CL | GTTTTTGTTGATACCAAGCCAAGTTAGAAGA**ATG**GGATTGAGAAGC**ATG**ACAAGACAATGTAGC |
| 24/30 | rev | CDR1 | pYD-VL-CL | GTTTTTGTTGATACCAAGCCAAGTTAGA**ATG**AACGGATTGAGAAGC**ATG**ACAAGACAATGTAGC |
| 24/31 | rev | CDR1 | pYD-VL-CL | GTTTTTGTTGATACCAAGCCAAGTT**ATG**AGAAACGGATTGAGAAGC**ATG**ACAAGACAATGTAGC |
| 24/32 | rev | CDR1 | pYD-VL-CL | GTTTTTGTTGATACCAAGCCAA**ATG**AGAAGAAACGGATTGAGAAGC**ATG**ACAAGACAATGTAGC |
| 24/33 | rev | CDR1 | pYD-VL-CL | GTTTTTGTTGATACCAAGC**ATG**GTTAGAAGAAACGGATTGAGAAGC**ATG**ACAAGACAATGTAGC |
| 24/34 | rev | CDR1 | pYD-VL-CL | GTTTTTGTTGATACCA**ATG**CAAGTTAGAAGAAACGGATTGAGAAGC**ATG**ACAAGACAATGTAGC |
| 25/26 | rev | CDR1 | pYD-VL-CL | GTTTTTGTTGATACCAAGCCAAGTTAGAAGAAACGGATTG**ATGATG**  TCTACAAGACAATGTAGC |
| 25/27 | rev | CDR1 | pYD-VL-CL | GTTTTTGTTGATACCAAGCCAAGTTAGAAGAAACGGA**ATG**AGA**ATG**TCTACAAGACAATGTAGC |
| 25/28 | rev | CDR1 | pYD-VL-CL | GTTTTTGTTGATACCAAGCCAAGTTAGAAGAAAC**ATG**TTGAGA**ATG**  TCTACAAGACAATGTAGC |
| 25/29 | rev | CDR1 | pYD-VL-CL | GTTTTTGTTGATACCAAGCCAAGTTAGAAGA**ATG**GGATTGAGA**ATG**  TCTACAAGACAATGTAGC |
| 25/30 | rev | CDR1 | pYD-VL-CL | GTTTTTGTTGATACCAAGCCAAGTTAGA**ATG**AACGGATTGAGA**ATG**  TCTACAAGACAATGTAGC |
| 25/31 | rev | CDR1 | pYD-VL-CL | GTTTTTGTTGATACCAAGCCAAGTT**ATG**AGAAACGGATTGAGA**ATG**  TCTACAAGACAATGTAGC |
| 25/32 | rev | CDR1 | pYD-VL-CL | GTTTTTGTTGATACCAAGCCAA**ATG**AGAAGAAACGGATTGAGA**ATG**TCTACAAGACAATGTAGC |
| 25/33 | rev | CDR1 | pYD-VL-CL | GTTTTTGTTGATACCAAGC**ATG**GTTAGAAGAAACGGATTGAGA**ATG**  TCTACAAGACAATGTAGC |
| 25/34 | rev | CDR1 | pYD-VL-CL | GTTTTTGTTGATACCA**ATG**CAAGTTAGAAGAAACGGATTGAGA**ATG**TCTACAAGACAATGTAGC |
| 26/27 | rev | CDR1 | pYD-VL-CL | GTTTTTGTTGATACCAAGCCAAGTTAGAAGAAACGGA**ATGATG**AGCTCTACAAGACAATGTAGC |
| 26/28 | rev | CDR1 | pYD-VL-CL | GTTTTTGTTGATACCAAGCCAAGTTAGAAGAAAC**ATG**TTG**ATG**AGC  TCTACAAGACAATGTAGC |
| 26/29 | rev | CDR1 | pYD-VL-CL | GTTTTTGTTGATACCAAGCCAAGTTAGAAGA**ATG**GGATTG**ATG**AGCTCTACAAGACAATGTAGC |
| 26/30 | rev | CDR1 | pYD-VL-CL | GTTTTTGTTGATACCAAGCCAAGTTAGA**ATG**AACGGATTG**ATG**AGC  TCTACAAGACAATGTAGC |
| 26/31 | rev | CDR1 | pYD-VL-CL | GTTTTTGTTGATACCAAGCCAAGTT**ATG**AGAAACGGATTG**ATG**AGC  TCTACAAGACAATGTAGC |
| 26/32 | rev | CDR1 | pYD-VL-CL | GTTTTTGTTGATACCAAGCCAA**ATG**AGAAGAAACGGATTG**ATG**AGCTCTACAAGACAATGTAGC |
| 26/33 | rev | CDR1 | pYD-VL-CL | GTTTTTGTTGATACCAAGC**ATG**GTTAGAAGAAACGGATTG**ATG**AGC  TCTACAAGACAATGTAGC |
| 26/34 | rev | CDR1 | pYD-VL-CL | GTTTTTGTTGATACCA**ATG**CAAGTTAGAAGAAACGGATTG**ATG**AGC  TCTACAAGACAATGTAGC |
| 27/28 | rev | CDR1 | pYD-VL-CL | GTTTTTGTTGATACCAAGCCAAGTTAGAAGAAAC**ATGATG**AGAAGC  TCTACAAGACAATGTAGC |
| 27/29 | rev | CDR1 | pYD-VL-CL | GTTTTTGTTGATACCAAGCCAAGTTAGAAGA**ATG**GGA**ATG**AGAAGCTCTACAAGACAATGTAGC |
| 27/30 | rev | CDR1 | pYD-VL-CL | GTTTTTGTTGATACCAAGCCAAGTTAGA**ATG**AACGGA**ATG**AGAAGCTCTACAAGACAATGTAGC |
| 27/31 | rev | CDR1 | pYD-VL-CL | GTTTTTGTTGATACCAAGCCAAGTT**ATG**AGAAACGGA**ATG**AGAAGCTCTACAAGACAATGTAGC |
| 27/32 | rev | CDR1 | pYD-VL-CL | GTTTTTGTTGATACCAAGCCAA**ATG**AGAAGAAACGGA**ATG**AGAAGCTCTACAAGACAATGTAGC |
| 27/33 | rev | CDR1 | pYD-VL-CL | GTTTTTGTTGATACCAAGC**ATG**GTTAGAAGAAACGGA**ATG**AGAAGCTCTACAAGACAATGTAGC |
| 27/34 | rev | CDR1 | pYD-VL-CL | GTTTTTGTTGATACCA**ATG**CAAGTTAGAAGAAACGGA**ATG**AGAAGCTCTACAAGACAATGTAGC |
| 28/29 | rev | CDR1 | pYD-VL-CL | GTTTTTGTTGATACCAAGCCAAGTTAGAAGA**ATGATG**TTGAGAAGC  TCTACAAGACAATGTAGC |
| 28/30 | rev | CDR1 | pYD-VL-CL | GTTTTTGTTGATACCAAGCCAAGTTAGA**ATG**AAC**ATG**TTGAGAAGC  TCTACAAGACAATGTAGC |
| 28/31 | rev | CDR1 | pYD-VL-CL | GTTTTTGTTGATACCAAGCCAAGTT**ATG**AGAAAC**ATG**TTGAGAAGC  TCTACAAGACAATGTAGC |
| 28/32 | rev | CDR1 | pYD-VL-CL | GTTTTTGTTGATACCAAGCCAA**ATG**AGAAGAAAC**ATG**TTGAGAAGCTCTACAAGACAATGTAGC |
| 28/33 | rev | CDR1 | pYD-VL-CL | GTTTTTGTTGATACCAAGC**ATG**GTTAGAAGAAAC**ATG**TTGAGAAGC  TCTACAAGACAATGTAGC |
| 28/34 | rev | CDR1 | pYD-VL-CL | GTTTTTGTTGATACCA**ATG**CAAGTTAGAAGAAAC**ATG**TTGAGAAGC  TCTACAAGACAATGTAGC |
| 29/30 | rev | CDR1 | pYD-VL-CL | GTTTTTGTTGATACCAAGCCAAGTTAGA**ATGATG**GGATTGAGAAGC  TCTACAAGACAATGTAGC |
| 29/31 | rev | CDR1 | pYD-VL-CL | GTTTTTGTTGATACCAAGCCAAGTT**ATG**AGA**ATG**GGATTGAGAAGCTCTACAAGACAATGTAGC |
| 29/32 | rev | CDR1 | pYD-VL-CL | GTTTTTGTTGATACCAAGCCAA**ATG**AGAAGA**ATG**GGATTGAGAAGCTCTACAAGACAATGTAGC |
| 29/33 | rev | CDR1 | pYD-VL-CL | GTTTTTGTTGATACCAAGC**ATG**GTTAGAAGA**ATG**GGATTGAGAAGCTCTACAAGACAATGTAGC |
| 29/34 | rev | CDR1 | pYD-VL-CL | GTTTTTGTTGATACCA**ATG**CAAGTTAGAAGA**ATG**GGATTGAGAAGCTCTACAAGACAATGTAGC |
| 30/31 | rev | CDR1 | pYD-VL-CL | GTTTTTGTTGATACCAAGCCAAGTT**ATGATG**AACGGATTGAGAAGC  TCTACAAGACAATGTAGC |
| 30/32 | rev | CDR1 | pYD-VL-CL | GTTTTTGTTGATACCAAGCCAA**ATG**AGA**ATG**AACGGATTGAGAAGCTCTACAAGACAATGTAGC |
| 30/33 | rev | CDR1 | pYD-VL-CL | GTTTTTGTTGATACCAAGC**ATG**GTTAGA**ATG**AACGGATTGAGAAGCTCTACAAGACAATGTAGC |
| 30/34 | rev | CDR1 | pYD-VL-CL | GTTTTTGTTGATACCA**ATG**CAAGTTAGA**ATG**AACGGATTGAGAAGCTCTACAAGACAATGTAGC |
| 31/32 | rev | CDR1 | pYD-VL-CL | GTTTTTGTTGATACCAAGCCAA**ATGATG**AGAAACGGATTGAGAAGCTCTACAAGACAATGTAGC |
| 31/33 | rev | CDR1 | pYD-VL-CL | GTTTTTGTTGATACCAAGC**ATG**GTT**ATG**AGAAACGGATTGAGAAGCTCTACAAGACAATGTAGC |
| 31/34 | rev | CDR1 | pYD-VL-CL | GTTTTTGTTGATACCA**ATG**CAAGTT**ATG**AGAAACGGATTGAGAAGCTCTACAAGACAATGTAGC |
| 32/33 | rev | CDR1 | pYD-VL-CL | GTTTTTGTTGATACCAAGC**ATGATG**AGAAGAAACGGATTGAGAAGCTCTACAAGACAATGTAGC |
| 32/34 | rev | CDR1 | pYD-VL-CL | GTTTTTGTTGATACCA**ATG**CAA**ATG**AGAAGAAACGGATTGAGAAGCTCTACAAGACAATGTAGC |
| 33/34 | rev | CDR1 | pYD-VL-CL | GTTTTTGTTGATACCA**ATGATG**GTTAGAAGAAACGGATTGAGAAGCTCTACAAGACAATGTAGC |
| 24/31/32 | rev | CDR1 | pYD-VL-CL | GTTTTTGTTGATACCAAGCCAA**ATGATG**AGAAACGGATTGAGAAGC**ATG**ACAAGACAATGTAGCTCTTTC |
| 89/90 | for | CDR3 | pYD-VL-CL | GCTGTTTACTACTGC**CATCAT**TACAACAATTGGCCATGGACTTTTGGTCAAG |
| 89/91 | for | CDR3 | pYD-VL-CL | GCTGTTTACTACTGC**CAT**CAA**CAT**AACAATTGGCCATGGACTTTTGGTCAAG |
| 89/92 | for | CDR3 | pYD-VL-CL | GCTGTTTACTACTGC**CAT**CAATAC**CAT**AATTGGCCATGGACTTTTGGTCAAG |
| 89/93 | for | CDR3 | pYD-VL-CL | GCTGTTTACTACTGC**CAT**CAATACAAC**CAT**TGGCCATGGACTTTTGGTCAAG |
| 89/94 | for | CDR3 | pYD-VL-CL | GCTGTTTACTACTGC**CAT**CAATACAACAAT**CAT**CCATGGACTTTTGGTCAAG |
| 89/95 | for | CDR3 | pYD-VL-CL | GCTGTTTACTACTGC**CAT**CAATACAACAATTGG**CAT**TGGACTTTTGGTCAAG |
| 89/96 | for | CDR3 | pYD-VL-CL | GCTGTTTACTACTGC**CAT**CAATACAACAATTGGCCA**CAT**ACTTTTGGTCAAG |
| 89/97 | for | CDR3 | pYD-VL-CL | GCTGTTTACTACTGC**CAT**CAATACAACAATTGGCCATGG**CAT**TTTGGTCAAG |
| 90/91 | for | CDR3 | pYD-VL-CL | GCTGTTTACTACTGCCAA**CATCAT**AACAATTGGCCATGGACTTTTGGTCAAG |
| 90/92 | for | CDR3 | pYD-VL-CL | GCTGTTTACTACTGCCAA**CAT**TAC**CAT**AATTGGCCATGGACTTTTGGTCAAG |
| 90/93 | for | CDR3 | pYD-VL-CL | GCTGTTTACTACTGCCAA**CAT**TACAAC**CAT**TGGCCATGGACTTTTGGTCAAG |
| 90/94 | for | CDR3 | pYD-VL-CL | GCTGTTTACTACTGCCAA**CAT**TACAACAAT**CAT**CCATGGACTTTTGGTCAAG |
| 90/95 | for | CDR3 | pYD-VL-CL | GCTGTTTACTACTGCCAA**CAT**TACAACAATTGG**CAT**TGGACTTTTGGTCAAG |
| 90/96 | for | CDR3 | pYD-VL-CL | GCTGTTTACTACTGCCAA**CAT**TACAACAATTGGCCA**CAT**ACTTTTGGTCAAG |
| 90/97 | for | CDR3 | pYD-VL-CL | GCTGTTTACTACTGCCAA**CAT**TACAACAATTGGCCATGG**CAT**TTTGGTCAAG |
| 91/92 | for | CDR3 | pYD-VL-CL | GCTGTTTACTACTGCCAACAA**CATCAT**AATTGGCCATGGACTTTTGGTCAAG |
| 91/93 | for | CDR3 | pYD-VL-CL | GCTGTTTACTACTGCCAACAA**CAT**AAC**CAT**TGGCCATGGACTTTTGGTCAAG |
| 91/94 | for | CDR3 | pYD-VL-CL | GCTGTTTACTACTGCCAACAA**CAT**AACAAT**CAT**CCATGGACTTTTGGTCAAG |
| 91/95 | for | CDR3 | pYD-VL-CL | GCTGTTTACTACTGCCAACAA**CAT**AACAATTGG**CAT**TGGACTTTTGGTCAAG |
| 91/96 | for | CDR3 | pYD-VL-CL | GCTGTTTACTACTGCCAACAA**CAT**AACAATTGGCCA**CAT**ACTTTTGGTCAAG |
| 91/97 | for | CDR3 | pYD-VL-CL | GCTGTTTACTACTGCCAACAA**CAT**AACAATTGGCCATGG**CAT**TTTGGTCAAG |
| 92/93 | for | CDR3 | pYD-VL-CL | GCTGTTTACTACTGCCAACAATAC**CATCAT**TGGCCATGGACTTTTGGTCAAG |
| 92/94 | for | CDR3 | pYD-VL-CL | GCTGTTTACTACTGCCAACAATAC**CAT**AAT**CAT**CCATGGACTTTTGGTCAAG |
| 92/95 | for | CDR3 | pYD-VL-CL | GCTGTTTACTACTGCCAACAATAC**CAT**AATTGG**CAT**TGGACTTTTGGTCAAG |
| 92/96 | for | CDR3 | pYD-VL-CL | GCTGTTTACTACTGCCAACAATAC**CAT**AATTGGCCA**CAT**ACTTTTGGTCAAG |
| 92/97 | for | CDR3 | pYD-VL-CL | GCTGTTTACTACTGCCAACAATAC**CAT**AATTGGCCATGG**CAT**TTTGGTCAAG |
| 93/94 | for | CDR3 | pYD-VL-CL | GCTGTTTACTACTGCCAACAATACAAC**CATCAT**CCATGGACTTTTGGTCAAG |
| 93/95 | for | CDR3 | pYD-VL-CL | GCTGTTTACTACTGCCAACAATACAAC**CAT**TGG**CAT**TGGACTTTTGGTCAAG |
| 93/96 | for | CDR3 | pYD-VL-CL | GCTGTTTACTACTGCCAACAATACAAC**CAT**TGGCCA**CAT**ACTTTTGGTCAAG |
| 93/97 | for | CDR3 | pYD-VL-CL | GCTGTTTACTACTGCCAACAATACAAC**CAT**TGGCCATGG**CAT**TTTGGTCAAG |
| 94/95 | for | CDR3 | pYD-VL-CL | GCTGTTTACTACTGCCAACAATACAACAAT**CATCAT**TGGACTTTTGGTCAAG |
| 94/96 | for | CDR3 | pYD-VL-CL | GCTGTTTACTACTGCCAACAATACAACAAT**CAT**CCA**CAT**ACTTTTGGTCAAG |
| 94/97 | for | CDR3 | pYD-VL-CL | GCTGTTTACTACTGCCAACAATACAACAAT**CAT**CCATGG**CAT**TTTGGTCAAG |
| 95/96 | for | CDR3 | pYD-VL-CL | GCTGTTTACTACTGCCAACAATACAACAATTGG**CATCAT**ACTTTTGGTCAAG |
| 95/97 | for | CDR3 | pYD-VL-CL | GCTGTTTACTACTGCCAACAATACAACAATTGG**CAT**TGG**CAT**TTTGGTCAAG |
| 96/97 | for | CDR3 | pYD-VL-CL | GCTGTTTACTACTGCCAACAATACAACAATTGGCCA**CATCAT**TTTGGTCAAG |
| 97 | for | CDR3 | pYD-VL-CL | GCTGTTTACTACTGCCAACAATACAACAATTGGCCATGG**CAT**TTTGGTCAAGGTACTAAGG |

**SUPPLEMENTARY TABLE 3** | Primer sequences for cloning.

| Name | | | Nucleotide sequence (5' --> 3') |
| --- | --- | --- | --- |
| PstI | for | pYD-VL-CL | CATTTTCAATTAAGACCATGAGATTTCCTTCAATTTTTACTGCAGTTTTATTC |
| SacI | rev | pYD-VL-CL | CTATTAACACTCTCCCCTGTTGAAGCTCTTTGTGACGGGCGAG |
| Fr2 | for | pYD-VL-CL | TGGTATCAACAAAAACCAGGTCAAGCTCCAAGATTATTG |
| CDR1 | rev | pYD-VL-CL | AGCCAAGTTAGAAGAAACGGATTGAGAAGCTCTACAAGAC |
| Fr3 | rev | pYD-VL-CL | GCAGTAGTAAACAGCGAAATCTTCGGATTGCAATG |
| CDR3 | for | pYD-VL-CL | CAACAATACAACAATTGGCCATGGACTTTTGGTCAAGG |
| pTT5 seq | for | pTT5-VL-CL | CTGCGCTAAGATTGTCAGT |
| I CLC to pTT5 | for | pTT5-VL-CL | GCTGCCTGTCAGACTGCTGGTGCTGATGTTCTGGATTCCCGCCAGCCTGAGCGAAATCGTCATGACTCAATC |
| II CLC to pTT5 | for | pTT5-VL-CL | GTTTAAACGGATCTCTAGCGAATTCGCGGTCGCCACCATGAAGCTGCCTGTCAGACTGC |
| Fr3 EcoRI mut | for | pTT5-VL-CL | CTGGTTCAGGTACTGAGTTCACCTTGACTATC |
| Fr3 EcoRI mut | rev | pTT5-VL-CL | GATAGTCAAGGTGAACTCAGTACCTGAACCAG |
| CLC to pTT5 | rev | pTT5-VL-CL | AGAGGTCGAGGTCGGGGGATCCTCATCAACACTCTCCCCTGTTGAAGC |
